# Society Meetings

**Published:** 1898-05

**Authors:** 


### SOCIETY MEETINGS.

THE Ohio state medical society will hold its next annual meeting at Columbus, May 4, 5 and 6, 1898, under the presidency of Dr. William H. Humiston, of Cleveland. An address on medicine will be delivered by Professor Hobart Amory Hare, M. D., of Philadelphia, and one on surgery by Professor Nicholas Senn, of Chicago. Reduced railway fares and hotel rates are offered, as well as a number of social functions, which, added to a fine scientific program, should secure a large attendance.

---

The Buffalo Academy of Medicine held meetings during the month of April as follows:

*Section on Surgery.*—Tuesday evening, April 5th, program: Two common forms of pyelitis, by Dr. J. Henry Dowd; Exhibition of specimens, by Dr. H. U. Williams; Lantern exhibitions of pathological specimens, also stereopticon views of diseases of the heart, by Dr. H. R. Gaylord, of Philadelphia.

*Section on Medicine.*—Tuesday evening, April 12th, program: State aid for the incipient consumptive, by Dr. J. H. Pryor; Myocardial changes; early diagnosis and prevention of fur ther development, by Dr. H. C. Buswell.

*Section on Pathology.*—Tuesday evening, April 19th, program: The scientific choice of fabrics for underwear, by Miss Mary Forster, of Newnham College, England; Some pathological findings in the blood; Exhibition of slides and drawings, by Dr. A. E. Woehnert.

*Section on Obstetrics and Gynecology.*—Tuesday, April 26th: The meeting of this section was postponed on account of the commencement of the medical department of the University of Buffalo.

---

The section of materia medica and therapeutics of the American Medical Association, that will meet at Denver, June 7 to 19, 1898, under the chairmanship of Dr. John V. Shoemaker, of Philadelphia, publishes an elaborate and interesting program. Dr. Willis G. Gregory, of Buffalo, will read a paper before the section, entitled, The relation of pharmacal legislation to pharmacal education.

---

The Lake Erie Medical Society held its regular meeting at Dayton, N. Y., Tuesday, April 26, 1898. The following was the program: Urethritis and stricture, Dr. B. C. Johnson, E. Utica street, Buffalo, N. Y.; Cystitis and irritable bladder, Dr. R. T. Rolph, Dunkirk, N. Y.; Prostatitis and senile hypertrophy, Dr. A. D. Lake, Gowanda, N. Y.; Pyelitis, Dr. S. S. Bedient, Little Valley, N. Y.

The Iowa State Medical Society will hold its forty-seventh annual meeting at Des Moines, May 18, 19 and 20, 1898, under the presidency of Dr. Edward Hornibrook, of Cherokee. The secretary is Dr. James W. Cokenower, of Des Moines.

---

The Michigan state board of health will celebrate the quarto centennial of its establishment at Detroit, August 9–11, 1898. On the invitation of Governor Pingree and the health department, Dr. Ernest Wende, health commissioner of Buffalo, will read a paper on the municipal prevention of disease. Extensive preparations have been made for the entertainment of guests, and the interest of the occasion will be greatly enhanced by the fact that the conference of state and provincial boards of health will be held at Detroit during the same period.

